# Predicting the Weathering Time by the Empty Puparium of *Sarcophaga peregrina* (Diptera: Sarcophagidae) with the ANN Models

**DOI:** 10.3390/insects13090808

**Published:** 2022-09-05

**Authors:** Xiangyan Zhang, Yang Bai, Fernand Jocelin Ngando, Hongke Qu, Yanjie Shang, Lipin Ren, Yadong Guo

**Affiliations:** 1 Department of Forensic Science, School of Basic Medical Sciences, Central South University, Changsha 410013, China; 2 School of Basic Medical Sciences, Central South University, Changsha 410013, China

**Keywords:** *Sarcophaga peregrina*, hydrocarbon, PMI, ANN

## Abstract

**Simple Summary:**

The weathering time of cuticular hydrocarbons from the puparium could assist in estimating the postmortem interval (PMI) of decomposed corpses. However, the composition of cuticular hydrocarbons in the puparium is complicated and has not been well studied for sarcophagid species. Therefore, we examined the compounds of *Sarcophaga peregrina* (Robineau-Desvoidy, 1830) at varying temperatures and used various machine learning models to predict the weathering time. The artificial neural network (ANN) model may be optimal for weathering time estimation.

**Abstract:**

Empty puparium are frequently collected at crime scenes and may provide valuable evidence in cases with a long postmortem interval (PMI). Here, we collected the puparium of *Sarcophaga peregrina* (Diptera: Sarcophagidae) (Robineau-Desvoidy, 1830) for 120 days at three temperatures (10 °C, 25 °C, and 40 °C) with the aim to estimate the weathering time of empty puparium. The CHC profiles were analyzed by gas chromatography-mass spectrometry (GC-MS). The partial least squares (PLS), support vector regression (SVR), and artificial neural network (ANN) models were used to estimate the weathering time. This identified 49 CHCs with a carbon chain length between 10 and 33 in empty puparium. The three models demonstrate that the variation tendency of hydrocarbon could be used to estimate the weathering time, while the ANN models show the best predictive ability among these three models. This work indicated that puparial hydrocarbon weathering has certain regularity with weathering time and can gain insight into estimating PMI in forensic investigations.

## 1. Introduction

There are approximately 3000 species of flesh flies (Diptera: Sarcophagidae) worldwide, excluding the Arctic and Antarctic [1]. These flies have high ecological, economic, agricultural, medical, and forensic importance [2]. The larvae can cause myiasis by infesting living and necrotic vertebrate tissues. In addition, flesh flies display a wide range of breeding strategies (including sarcosaprophagy, coprophagy, predation, and kleptoparasitism) due to the larvae being omnivorous [3]. Due to their association with corpses and carrion, flesh flies can be used to estimate the time since death (also known as postmortem interval (PMI)) in homicides, animal maltreatment, and senior/child neglect [4,5,6]. Immature life stages (including eggs, larvae, and pupae) and empty puparium are frequently collected from decomposed corpses, which are the insect evidence with the highest incidence at the death scene [7]. However, the empty puparium may be the only entomological evidence when the decomposition of the corpse has become highly advanced, even years later [8,9,10]. Thus, determining the weathering time of empty puparium would be of great significance for estimating PMI [11,12].

The puparium of flesh flies, generated on the surface of the pupal body without molting during pupation, has a similar chemical composition as the cuticle of the third instar [13,14]. The cuticle is covered by an epicuticular lipid wax layer, mainly composed of hydrocarbons [15]. Cuticular hydrocarbons (CHCs), consisting of n-alkanes, alkenes, monomethyl alkanes, and polymethyl alkanes [16,17,18], primarily function to thwart the insect from environmental stress and serve as a pheromone [17,19,20]. Using CHCs with ANN Moore et al. (2016), we are able to obtain 87% accuracy in estimating the age of larvae of *Calliphora vicina* and *Calliphora vomitoria* (Diptera: Calliphoridae) [19]. In addition, utilizing CHCs and ANN shows great potential for adult age estimates for *Lucilia sericata*, *C. vicina*, and *C. vomitoria* [20]. Moreover, hydrocarbon compounds are highly stable even when samples are preserved for hundreds of years [21]. *Chrysomya megacephala* (Fabricius, 1794) (Diptera: Calliphoridae) exhibited a significant association between the abundance of C22, C24, and C26 (n-alkanes with 22, 24, and 26 carbon atoms, the same as below) and weathering time of puparium [11]. Moreover, comparing puparium contents of *Hydrotaea aenescens* (Wiedemann, 1830) (Diptera: Muscidae) in a 15-year difference indicated that there were significant differences in the composition of puparium, especially the length of n-alkanse and ester chains [9]. The differences can also be used for species identification on groups of medical or agricultural insects [22,23]. Although the chemical composition of puparium varies from species to species, little is known about the weathering time of CHCs in the Sarcophagidae family. Therefore, the relationship between the weathering model and the hydrocarbon profile of puparium in Sarcophagidae needs to be further studied. Moreover, it is of great significance to explore the weathering time of CHCs for estimating the long-term PMI [12].

In this study, we selected *Sarcophaga peregrina* (Robineau-Desvoidy, 1830) (Diptera: Sarcophagidae), a synanthropic fly worldwide [24]. It is regarded as a carrier of diseases, and some are recorded as being parasitic [25]. We collected the puparium of *S. peregrina* for 120 days under three temperatures (10 °C, 25 °C, and 40 °C). This range of temperature almost encompasses the common development temperature of *S. peregrina*. The profile of CHCs was then identified using Gas Chromatography–Mass Spectrometry (GC-MS). GC-MS is an effective method for qualitative and quantitative analysis of organic matter. Finally, we compared three kinds of regression algorithms (PLS, SVR, and ANN) by metrics (R2, RMSE, MSE, and MAE) to find which algorithm was more suitable for the weathering time estimate. 

## 2. Materials and Methods

### 2.1. Samples Collection

*S. peregrina* originated from a laboratory colony initiated from pig carcasses in Changsha city (28º12’ N, 112º58’ E), Hunan province, China. The insects were kept in the laboratory for more than three years. Each year, wild flies were mixed with laboratory flies to reduce gene and phenotypic differences between laboratory and wild flies. The flies used in this study were reared in 2020 [26]. The adults were raised in a rearing cage (35 × 35 × 35 cm^3^) at a temperature of 25.0 ± 1.0 °C) with 70% humidity and a photoperiod of 12:12 Light: Dark cycle. They were fed milk powder mixed with sugar and freshwater with degreasing cotton in 12 cm diameter dishes.

For sample collection, fresh swine lung was employed to induce larviposition. Larvae larviposited within 2 h were collected and reared in a plastic bowl (18 cm diameter, 5 cm height) with a moderate amount of pork lung. The bowl was put into a box (25 × 25 × 12 cm^3^) with 2 cm of silver sand covering the bottom until pupation. We collected the puparium of *S. peregrina* that emerged on the first day as experimental samples (the first day was called 0 day in this study). Then, the puparium was divided into three groups and placed at different temperatures (10 °C, 25 °C, and 40 °C). 

Three puparium were collected every day of the first five days, every five days from day 5 to day 40, and every ten days from day 40 to day 120. A total of 63 puparium at each temperature were collected. All samples were stored separately in 1.5 mL cryovials and stored at −80 °C until analysis.

### 2.2. Gas Chromatography-Mass Spectrometry (GC-MS) Analysis

The puparium was cleaned in ultrapure water and blotted dry with filter paper. Then, each individual was immersed in 1 mL hexane with the internal standard tetracosane (C24, 0.25 μg/mL, LGC) in a 2 mL glass vial at room temperature for an hour. Next, a syringe filter transferred the immersed liquid with a 0.45 μm aperture nylon membrane. Afterwards, the liquid was dried under vacuum and dissolved in 100 μL hexane for GC-MS analysis.

GC-MS (Agilent Technologies, Santa Clara, CA, USA, 7890B-5977A GC/MSD), with a DB-5MS capillary column (30 m × 0.25 mm × 0.25μm), was used for the CHCs analysis. In total, 1μL of liquid was injected splitless at 250 °C. The oven temperature program was initiated at 50 °C for 2 min then ramped to 200 °C at 25 °C/min, then to 260 °C at 6 °C/min, to 300 °C at 3 °C/min and finally, it was held for 15 min. The temperature of the GC-MS interface was 280 °C. The carrier gas used ultrapure helium with a pressure of 11.3 psi. Electron impact mode was set at 70 eV, and ion source temperature was set at 230 °C. The n-alkanes mix from heptane to tetracontane (C7–C40, 1 μg/mL, O2SI) resolved in 1 mL hexane was used as an external standard.

MSD ChemStation Data Analysis F.01.03 was used to integrate the peak areas, and only compounds with a consistent peak area percentage above 0.5% were included. Hydrocarbons were identified using a library search (NIST14), Kovats Index based on external standards, and literature [27,28,29,30,31,32,33]. The recognition of homologous peaks (retention index) was more important than chemical identification in the case [34].

### 2.3. Regression Algorithms 

Data set: We randomly selected 80% of the samples to train the model, with the remaining 20% of the samples used to validate the performance and accuracy of the estimation model. The percentage composition of CHCs was regarded as input data matrix X, and the weathering time was regarded as the Y value. The missing value was set as zero. The input data set size is shown in Table 1. The normalization data was used to train the machine learning models.

Metrics: The R Squared (R^2^), root mean squared error (RMSE), mean squared error (MSE), and mean absolute error (MAE) of the model were calculated to evaluate the performance and accuracy of the SVR model. Detail equations could be found in the previous study [35].

Partial Least Squares (PLS): PLS is a usual multivariate statistical regression method. The number of the principal components was set in (1, 10), and the max iters were set at 10. The best parameters were selected by grid search and cross-validation. The variable importance of projection (VIP) parameter was used to describe the contribution of variables to the model [36]. In addition, orthogonal projections to latent structures discrimination analysis (OPLS-DA) were used to visualize the difference in hydrocarbon at different temperatures. PLS and OPLS-DA were conducted in SIMCA 14.1 and Python 3.7.4.

Support Vector Regression (SVR): SVR is a vital application branch model of support vector machines (SVM) that can be employed for regression problems [37,38]. The input data vectors are mapped to a multi-dimensional eigenspace using a three-order kernel function called radial basis function kernel (RBF). The main hyper-parameters of SVR are the penalty error (C∈(1, 1000)) and the kernel index (γ∈(0.0005, 0.5)). The best parameters were selected by grid search and cross-validation. The models were conducted in Python 3.7.4.

Artificial Neural Network (ANN): ANN is a complex network of interconnected processing units. Here, we used a six-layer network conducted in Python 3.7.4. The first layer is Batch Normalization, followed by a layer with 61 units, a dropout layer, a layer with 97 units, a dropout layer, and a layer with 13 units. The linear rectification function (ReLU) was chosen as the activation function. The adaptive moment estimation (Adam) was selected as the optimizer. The learning rate was between 0.0001 and 0.1, epochs were between 10 and 1000, and batch sizes were between 16 and 40. The best parameters were selected by grid search and cross-validation.

## 3. Results

The CHCs profile of *S. peregrina* puparium of this study is shown in Tables S1–S3. At three temperatures (10 °C, 25 °C, 40 °C) and 120 days after eclosion, 49 CHCs associated with the puparium were identified by GC-MS analysis, including 21 n-alkanes, 20 branched alkanes, 2 alkenes, and 6 unknown compounds with the carbon chain length between C10–C33. 

### 3.1. The OPLS-DA Models of S. peregrina Puparium Classified by Temperatures

**Figure 1:**
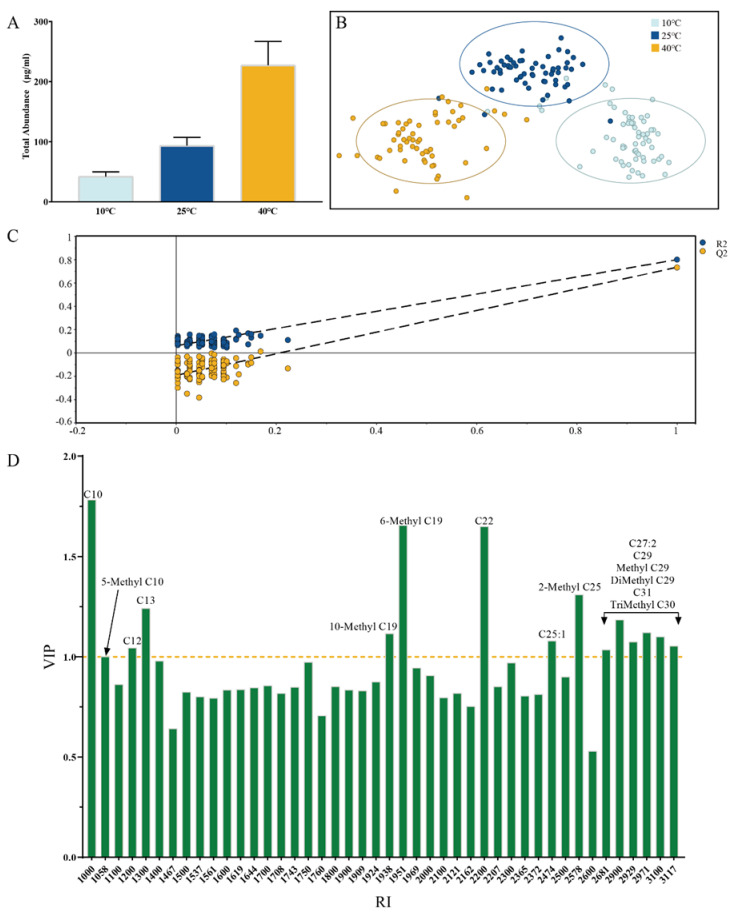
**(A**): Total abundance of *S. peregrina* puparia hydrocarbons in three temperatures (10 °C, 25 °C, 40 °C). (**B**): OPLS−DA of the various compositions of *S. peregrina* puparia. (**C**): The result of response permutation testing. (**D**): The hydrocarbons with VIP of OPLS−DA model. VIP > 1 regarded the hydrocarbons as significant.

As shown in Figure 1A, the abundance of the hydrocarbons at higher temperatures can be detected more than at lower temperatures in the 120 days after eclosion. To visualize the difference in the hydrocarbon distribution of *S. peregrina* at different temperatures, OPLS-DA models were built, as shown in Figure 1B, with R2X 87.5%, R2Y 77.3%, and Q2Y 70.9%. The samples could be clustered into three parts according to the temperature (10 °C, 25 °C, 40 °C). The parameters above show that the model has reasonable explanatory and prediction rates. The intercept of Q2 on the Y-axis of response permutation testing (Figure 1C) is less than 0, suggesting the model is not over-fitting. Figure 1D presents an overview of different hydrocarbons’ variable importance of projection (VIP). There are 15 hydrocarbons with VIP > 1 (Table S4), especially for C10, 6-Methyl C19, and C22, which significantly differ at different temperatures. In summary, these results show a noticeable difference in hydrocarbon in the puparium at different temperatures.

### 3.2. The PLS Model of S. peregrina Puparium

According to the grid search and cross-validation, the number of principal components and max iters were picked, as shown in Table 2. In the three groups (10 °C, 25 °C, and 40 °C), max iters were set as 10. The number of principal components was set as 9, 2, and 3 for the three groups. As a result, the R2 of the PLS model is 0.86 in the training set, while only 0.55 in the validation set (10 °C group in Table 3). In the 25 °C group, the goodness of fit is even worse, only 0.53 in the training set and 0.7 in the validation set. In the 40 °C group, the goodness of fit is relatively better than other groups, with 0.71 in the training set and 0.64 in the validation set.

### 3.3. The SVR Model of S. peregrina Puparium

In SVR, the kernel transforms the data to a higher-dimensional space where data will be linearly separable. The popular kernel functions include linear kernel, polynomial function, sigmoid kernel, and Gaussian radial basis function (RBF) kernel [39]. In this study, we chose RBF as the kernel for the result of regression used. RBF is the best in the kernels above. The best hyper-parameters of SVR are shown in Table 4; they were selected according to grid search and cross-validation results. In the 10 °C group, C was set at 310, and γ was set at 0.045; In the 25 °C group, C was set at 499, γ was set at 0.293; In the 40 °C group, C was set at 200, γ was set at 0.1. The metrics of R^2^, RMSE, and MAE are shown in Table 3. In the 10 °C group, the R^2^ of the SVR model is 0.98 in the training set, while only 0.57 in the validation set. In the 25 °C group, the R^2^ is 0.91 in the training set and 0.4 in the validation set. In the 40 °C group, the goodness of fit is relatively better than other groups, with 1 in the training set and 0.66 in the validation set.

### 3.4. The ANN Model of S. peregrina Puparium

We constructed the ANN model with six hidden layers. The best parameters were selected by grid search and cross-validation, shown in Table 5. The batch size in all groups was set at 32. In the 10 °C group, the learning rate was set at 0.001, and epochs were set at 1000. In the 25 °C group, the learning rate was set at 0.01, and epochs were set at 100; In the 40 °C group, the learning rate was set at 0.0001, and epochs were set at 1000. The metrics of R^2^, RMSE, and MAE are shown in Table 3. In the 10 °C group, the R^2^ of the ANN model is 0.96 in the training set and 0.81 in the validation set. In the 25 °C group, the R^2^ is 0.79 in the training set and 0.61 in the validation set. In the 40 °C group, R^2^ is 0.88 in the training set and 0.76 in the validation set.

## 4. Discussion

Empty puparium are widely found in the advanced stage of cadaver decomposition; and sometimes they may be the only entomological evidence at the crime scene [9,40]. Therefore, the weathering time of puparium can provide more valuable evidence for long-term PMI estimation [40]. Unfortunately, the methods for estimating the weathering time of puparium have been rarely reported. In this study, the results indicated that the hydrocarbon of *S. peregrina* puparium changed with weathering time, which is consistent with that of Zhu (2007), who found that puparial hydrocarbons of *C. megacephala* could be used as an indicator of PMI [11]. However, the weathering phenomenon under different constant temperatures has not been documented. The results of this study indicate that the abundance of the hydrocarbons dropped dramatically with weathering time at lower temperatures. As reported by Zhu et al., an improved exponential function could be used to simulate the weathering time of puparial hydrocarbons during the first 50 days [41]. It is somewhat surprising that the content of the hydrocarbons escalated with time at high and moderate temperatures. It is difficult to explain this result, but it might be related to microorganisms, as the experiment did not exclude microbial interference. Furthermore, this increasing tendency was also found by Zhu et al.(2007) [11]. They suspected that the cleavage of the branched alkanes with high molecular weight might be a crucial reason for the change in hydrocarbon content, because tertiary carbon radicals are highly reactive and can be cleaved at branched sites. According to the complexity of the abundance of hydrocarbons and weathering time, estimating PMI utilizing a single compound is inappropriate. Therefore, seeking the appropriate multiple regression method is a crucial point. As our study showed, the PLS, SVR, and ANN models based on *S. peregrina* puparial hydrocarbons could be used to estimate the weathering time of the empty puparium. The PLS, SVR, and ANN models are the most commonly used regression models [39]. The PLS model could be traced back to Herman Wold’s nonlinear iterative partial least squares algorithm [42]. Comparing the metrics of these three models, the PLS models presented the weakest imitative effect, with the R^2^ of the total sets not exceeding 0.5 and the RMSE not being less than 19 days. In contrast, the ANN models showed a better predictive performance with the RMSE not more than 19 days. The metrics of the total sets of the SVR models may be superior to the ANN models in the group of 25 °C and 40 °C. In the validation sets, however, their metrics are worse than those of the ANN models. Therefore, we concluded that among these three models, the ANN model provides the most accurate estimation of weathering time. Though most of the R^2^ did not reach 0.9, the result was more precise than others reported by utilizing the empty puparium to estimate the PMI [43].

In addition, the temperature has a significant impact on the changing patterns of hydrocarbons. At different temperatures, the difference in the hydrocarbon content of the puparium was evident. A previous study on *Chrysomya rufifacies* (Macquart, 1843) shows a difference in the weathering rate between seasons: the colder the season, the lower the weathering rate [41]. The study of biodegradation of crude oil in Arctic seashore sediments [44] drew the same conclusion. Here, a possible explanation might be that higher temperatures increase biodegradation. According to our study, a larger concentration of hydrocarbons could be discovered at a higher temperature in the puparium. This result may be explained by the fact that higher temperature has a more significant influence on the surface layer than the deeper structure, and it accelerates the weathering of the surface layer, making us detect both superficial and deep hydrocarbons. Microbiological degradation is another possible alternative reason [44]. Most microbial communities degraded more medium-chain (C14–C30) n-alkanes than long-chain (C31–C34) or short-chain (C10–C13) n-alkanes (except for H16S, which displayed high degradation rates against long-chain n-alkanes of C31 to C34 [45]).

Before our results can be translated into a proper forensic technique for estimating the PMI, more attention should be focused on the factors influencing the cuticular hydrocarbon composition of empty puparium. The puparium storage temperature is one crucial factor. Our results could be helpful for cases at room temperature, which coincides with slight temperature fluctuations. However, when considering cases in the field under fluctuating temperature, our models may give a great error for PMI estimation. *Lucilia ochricornis* and *Lucilia purpurascens* show different developmental rates between field and laboratory conditions [46], which means fluctuating temperature may affect the composition of CHCs of empty puparium. Future work needs to focus on how fluctuating temps will impact the chemical profiles. Moreover, the microorganisms, wind, rain, solar irradiance, and other environmental factors can also affect the weathering rate of CHCs. Using CHCs to estimate PMI accurately is still a long way off.

## 5. Conclusions

The results present in this preliminary study revealed that CHC profiles and temperature combined with the ANN model could be a potential tool for puparium weathering time, which could serve as an indication of possible PMI in forensic casework. Future studies will include more of the influencing factors, such as microorganisms and humidity, to better understand the puparium weathering.

## Figures and Tables

**Table 1:** Input dataset size.

Group	Training Set	Validation Set
10 °C	(50, 49)	(12, 49)
25 °C	(50, 49)	(12, 49)
40 °C	(46, 49)	(12, 49)

**Table 2:** The optimum parameter of the PLS model.

Group	Number of Principal Components	Max Iters
10 °C	9	10
25 °C	2	10
40 °C	3	10

**Table 3:** The metrics of ANN, SVR, and PLS model.

Group	Model	Training Set				Validation Set				Total Set			
		R^2^	RMSE	MSE	MAE	R^2^	RMSE	MSE	MAE	R^2^	RMSE	MSE	MAE
10 °C	ANN	0.96	7.6	57.78	5.39	0.81	17.9	320.36	12.44	0.94	9.5	90.32	5.76
	SVR	0.98	6.22	38.73	2.08	0.57	20.94	438.61	18.92	0.92	11.07	122.58	5.61
	PLS	0.86	14.27	203.76	10.84	0.55	27.92	779.3	22.89	0.78	18.01	324.44	13.36
25 °C	ANN	0.79	17.22	296.5	11.25	0.69	23.19	537.77	15.88	0.77	18.63	347.09	12.22
	SVR	0.91	11.89	141.38	3.79	0.43	24.23	587.13	19.11	0.84	15.32	234.84	7
	PLS	0.53	25.95	673.16	20.18	0.57	27.2	739.58	20.27	0.54	26.21	687.08	20.2
40 °C	ANN	0.88	13.5	182.32	10.21	0.76	15.88	252.06	12.4	0.86	14.03	196.75	10.66
	SVR	1	0.66	0.44	0.19	0.66	21.38	457.18	16.21	0.93	9.74	94.94	3.51
	PLS	0.71	21.02	441.89	16.59	0.64	19.39	376.14	14.44	0.7	20.7	428.29	16.15

**Table 4:** The optimum parameter of the SVR model.

Group	C	γ
10 °C	310	0.045
25 °C	499	0.293
40 °C	200	0.1

**Table 5:** The optimum parameter of the ANN model.

Group	Learning Rate	Epochs	Batch Size
10 °C	0.001	1000	32
25 °C	0.01	100	32
40 °C	0.0001	1000	32

## Data Availability

The data and code presented in this study are available on request from the corresponding author.

## Supplementary Materials

The following supporting information can be downloaded at: https://www.mdpi.com/article/10.3390/insects13090808/s1, Table S1–S3: CHCs profile of *S. peregrina* at different weathering times under 10 °C, 25 °C, 40 °C. Table S4: The hydrocarbons with VIP > 1 of OPLS-DA and PLS model.
